# Physical risk factors for adolescent neck and mid back pain: a systematic review

**DOI:** 10.1186/s12998-018-0206-y

**Published:** 2018-09-24

**Authors:** Brigitte Wirth, Tobias Potthoff, Sandra Rosser, Barry Kim Humphreys, Eling D. de Bruin

**Affiliations:** 1 Integrative Spinal Research, Department of Chiropractic Medicine, University Hospital Balgrist, Forchstr. 340, 8008 Zurich, Switzerland; 2 Department of Health Sciences and Technology, Institute of Human Movement Sciences and Sport, ETH Zurich, Zurich, Switzerland; 3 Department of Neurobiology, Care Sciences and Society, Karolinska Institutet, Huddinge, Sweden

**Keywords:** Adolescent, Mid back pain, Neck pain, Systematic review

## Abstract

**Background:**

Besides low back pain (LBP), also neck pain (NP) and mid back pain (MBP) are common health issues in adolescence. Psychological factors are regarded as main risk factors for spinal pain in adolescence, but recent studies suggest that the importance of physical factors might be underestimated. The purpose of this study was to summarize the results of studies on physical risk factors for adolescent NP and MBP.

**Methods:**

Cross-sectional and prospective English studies on NP and MBP in adolescents aged 10 to 18 were searched by a professional librarian in Medline (OvidSP), Premedline (PubMed), EMBASE, Cochrane, CINAHL, PEDro and PsycINFO up to October 2016. Studies that were restricted to self-report via questionnaires were excluded.

**Results:**

Eight cross-sectional studies could be included in this review. Some aspects of sagittal alignment in sitting (increased lumbar lordosis) and standing (anteroposition of the head, sway-back posture) were associated with NP. Study comparability was impeded by inconsistent definitions of NP and MBP and a wide variety of outcome measures.

**Conclusions:**

This systematic review indicates that prospective studies using a consistent definition of NP and MBP are needed. Such studies might further investigate sagittal alignment in sitting and standing as possible risk factors for NP and MBP in adolescence using a consistent terminology for the outcomes and longitudinal research designs.

**Electronic supplementary material:**

The online version of this article (10.1186/s12998-018-0206-y) contains supplementary material, which is available to authorized users.

## Introduction

In adolescents between 15 and 19 years, low back pain (LBP) and neck pain (NP) rank within the top ten for the years lived with disability worldwide and rank higher than some well-recognized health problems of adolescence such as alcohol and drug abuse [1]. In a Norwegian study with 7373 adolescents between 13 and 19 years, neck/shoulder was the most often affected location of musculoskeletal pain [2]. There is some evidence that also the importance of mid back pain (MBP) [thoracic spine pain (TSP)] should not be underestimated in adolescence: in contrast to adulthood, where MBP incidence and prevalence is considerably lower than that of LBP and NP [3, 4], MBP incidence is similar to that of LBP and NP in children and adolescents [4]. MBP prevalence even outnumbers LBP prevalence at the age of 9 and equalizes it at the age of 15 [5, 6]. These numbers are of particular relevance as several studies have shown that pain experience in childhood and adolescence impacts pain experience later in life [7, 8]. For LBP for example, an eight-year follow up from adolescence to adulthood showed that the risk of LBP in adulthood was fourfold when LBP was reported in adolescence [9]. The same seems to apply for MBP. From 58 children with persisting non-specific LBP or MBP, 90% of the children with MBP and 55% of those with LBP reported pain after skeletal maturity [10]. Thus, not only LBP, but also NP and MBP are common in childhood and adolescence and affect health in adulthood. Whether the underlying mechanisms for adolescent NP and MBP are physiological, psychological, behavioral, genetic or a combination of these is unknown [1]. Commonly, psychosocial factors and psychological distress are regarded as main risk factors for spinal pain in adolescence, while the relevance of physical (e.g. posture, mobility, endurance, strength, anthropometric measures) is less clear [1] and was suggested to be less important [11] . Nevertheless, a recent study reported that psychosomatic symptoms were most strongly associated with the prevalence of adolescent spinal pain, but these were followed by factors from the physical and psychosocial domains, and the role of lifestyle factors, such as physical activity, was limited [12]. Consequently, the authors suggested that the importance of physical risk factors for non-specific adolescent spinal pain may have been underestimated so far. Similarly in adults, the majority of studies investigated psychosocial or work related risk factors [13] and focused on LBP [14]. One systematic review on physical risk factors for neck/shoulder pain (NSP) reported inconclusive evidence for muscle strength, muscle endurance and cervical spine mobility as possible risk factors for NSP, due to a limited number of studies (*N* = 3). The goal of this systematic review was to summarize the results of studies on physical risk factors for NP and MBP in adolescents between 10 and 18 years including solely studies that used quantifiable measures beyond questionnaires.

## Literature search methods

### Search strategy

A structured review protocol was a priori developed by three authors (BW, TP, EdB). The search strategies were generated with the support of a librarian from the local university library (Additional file 1). The databases Medline (OvidSP), Premedline (PubMed), EMBASE, Cochrane, CINAHL, PEDro and PsycINFO were searched up to and including September 25, 2015 and again on October 24, 2016. The search was not restricted to NP and MBP because this review was part of a larger project that also investigated physical risk factors for LBP. At this stage, the search was also not restricted to physical factors as these might have been secondary outcomes of studies on psychosocial factors. Medical subheadings (MeSH) were used as search terms. In addition, to find the most recent publications that have not yet been linked with MeSH, keywords were also searched for in the title or abstract.

### Inclusion and exclusion criteria

This review included English language studies that were cross-sectional, prospective or retrospective and investigated back pain in adolescents between 10 and 18 years. Age was limited to this range because pubertal development, starting at 9.5 years for girls and at 10 years for boys [15], might be a risk factor for back pain in the young [16]. Studies that covered a wider age range were included only if the mean age of the group(s) under investigation was within the limits of this review. Another inclusion criterion was that the studies were not restricted to questionnaire-based outcomes, but reported on quantitative measures. Furthermore, studies were excluded if they investigated exclusively lifestyle factors (computer activities, school bag weight, body weight or sport activities), focused on particular populations such as athletes or disabled children or on pain associated with specific pathologies (scoliosis, Scheuermann’s disease, spondylolisthesis, disc degeneration, hypermobility, coccydynia, fibromyalgia, posttraumatic or postoperative back pain, radiographic studies).

### Study selection

Titles and abstracts of the articles were screened by two authors (BW and TP) according to the inclusion and exclusion criteria listed above. In a second step, the full text of the remaining articles was screened for eligibility by the same two authors. The full text was also screened if no abstract was available or eligibility was unclear based on the text of the abstract. Two consensus meetings helped to resolve any discrepancies in terms of eligibility. In a last step, studies that focused on neck and/or mid back were selected for this review.

### Quality assessment

Two authors (BW and SR) assessed the quality of the selected studies based on the “Critical appraisal form for quantitative studies” [17]. Because no intervention was investigated, the questions referring to any intervention were removed as done in a comparable review [18]. Instead, a question on estimates of random variability of data was added from the Downs and Black checklist [19] and two questions on biases [18] and on the adequate description of the assessments [18, 19] were included. The assessment form is shown in Additional file 2. All questions were either answered by YES (= 1 point) or NO (= 0 points) except for the question on biases where the scoring was reversed. As the question addressing drop-outs was only applicable to prospective studies, the total quality score was maximally 14 points for cross-sectional and retrospective studies and 15 points for prospective studies. After individual rating, a consensus meeting was held to clarify possible disagreements. The agreement of the two ratings was calculated by Cohen’s kappa using IBM SPSS Statistics 21. A study’s quality was regarded as moderate to high if it reached at least 60% of the maximum score [20].

### Data extraction

From each article, one author (TP) extracted information about the study design, number, age and gender of participants, the physical factors that were investigated, the assessments and tests that were used, and the main results. A second author (BW) double-checked these data.

The reporting of this systematic review followed the PRISMA guidelines [21] (Additional file 3).

## Results

### Study selection and quality appraisal

**Fig. 1:**
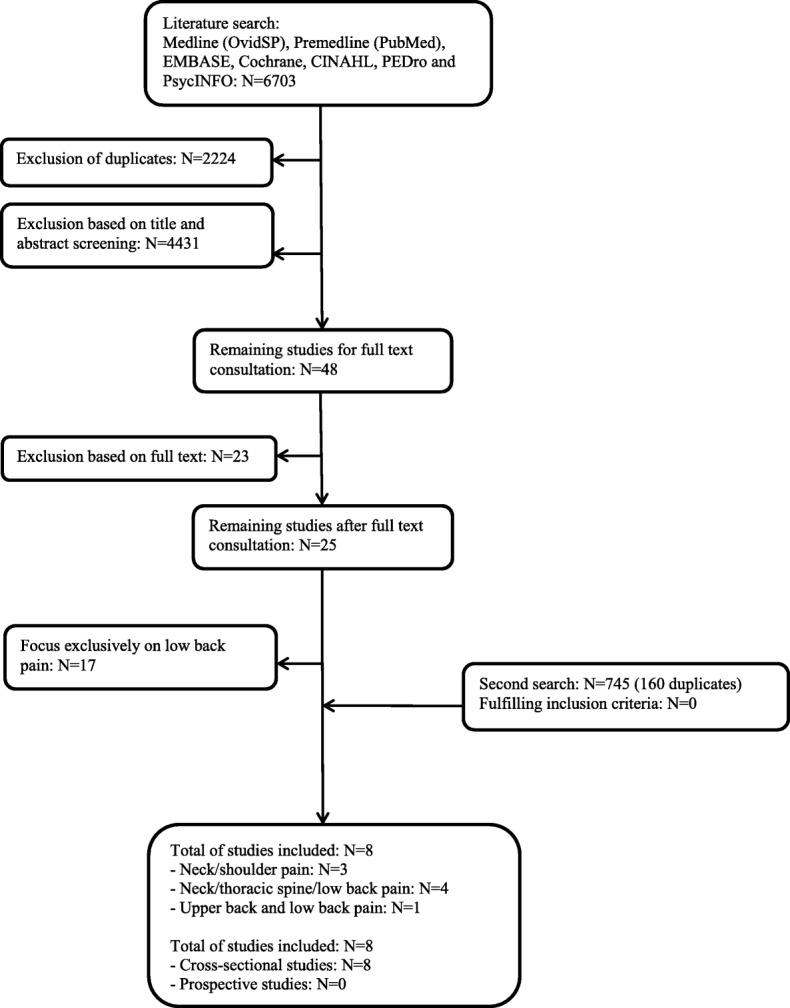
Flowchart of study search, eligibility and inclusion

The flow chart in Fig. 1 illustrates the study selection process that resulted in a total of eight cross-sectional studies to be included in this review [22–29].

Three studies focused on NSP [26–28], four studies separately investigated pain in the neck, the thoracic spine and in the lower back [23–25, 29], and one study focused on upper and lower back pain [22]. After the consensus meeting, the two reviewers agreed on the scores of all articles, expressed in a resulting κ value of 1.00 (*p* < 0.001). The mean quality score of the studies was 10.3 ± 1.8 (range: 8 to 13 points out of 14 points). One cross-sectional study did not reach moderate quality level according to the pre-assessment adopted criterion from the PEDro guidelines (Tables 1 and 2).

**Table 1:** Summary of study characteristics of included studies

Author, year	Title	Pain localization	Pain assessment: questionnaire	Study design	Participants: number (m/f) age	Investigated physical factors	Main results	Quality score
Cudre-Mauroux et al., 2006 [22]	Relationship between impaired functional stability and back pain in children: an exploratory cross-sectional study	Upper and lower back	Week prevalence	Cross-sectional	*N* = 125 (57/68) Median age = 10 years	Functional stability (Matthiass Test using a new scoring system)	No association between Matthiass test and upper back pain in the last week.	< 60%/14
Dolphens et al., 2012 [23]	Sagittal standing posture and its association with spinal pain.	Neck, thoracic spine, low back	Lifetime and one-month prevalence, concomitant doctor visit	Cross-sectional	*N* = 1196 (639/557) Boys: 12.6 ± 0.5 years Girls: 10.6 ± 0.5 years	Global sagittal alignment (pelvic displacement, trunk lean angle, body lean angle) and local spinopelvic parameters (e.g. number of vertebrae in the lumbar lordosis, vertebral level of apex, pelvic orientation in the sagittal plane) in habitual standing using digital images, inclinometry and accelerometry.	Neck pain: Boys: Positive association between lifetime prevalence of neck pain and anteroposition of the head (smaller craniovertebral angle) and increased trunk lean angle (*R*^2^ = 0.03). Positive association between month prevalence of neck pain and increased trunk lean angle (*R*^2^ = 0.06). Girls: Positive association between lifetime prevalence of doctor visit and anteroposition of the head (*R*^2^ = 0.04). Mid back pain: Boys: Positive association between doctor visits and increased trunk lean angle (*R*^2^ = 0.05). Girls: No significant association.	12/14
Dolphens et al., 2013 [24]	Classification system of the normal variation in sagittal standing plane alignment. A study among adolescent boys.	Neck, thoracic spine, low back	Lifetime and one-month prevalence, concomitant doctor visit	Cross-sectional	*N* = 639 (639/0) 12.6 ± 0.5 years	Global sagittal alignment (pelvic displacement, trunk lean angle, body lean angle) and local spinopelvic parameters (e.g. number of vertebrae in the lumbar lordosis, vertebral level of apex, pelvic orientation in the sagittal plane) in habitual standing using digital images, inclinometry and accelerometry.	Cluster analysis: 3 clusters of global alignment: neutral, sway-back, leaning-forward. Sway-back (large trunk lean angle and large body lean angle) significantly associated with lifetime prevalence of neck pain. Global posture category accounts for 2% in lifetime prevalence of neck pain and for 4% in month prevalence of neck pain. No association of spinal pain measures and local spinopelvic parameters.	11/14
Dolphens et al., 2014 [25]	Classification system of the sagittal standing alignment in young adolescent girls	Neck, thoracic spine, low back	Lifetime and one-month prevalence, concomitant doctor visit	Cross-sectional	*N* = 557 (0/557) 10.6 ± 0.5 years	Global sagittal alignment (pelvic displacement, trunk lean angle, body lean angle) and local spinopelvic parameters (e.g. number of vertebrae in the lumbar lordosis, vertebral level of apex, pelvic orientation in the sagittal plane) in habitual standing using digital images, inclinometry and accelerometry.	3 postural subtypes No association between posture clusters and spinal pain measures.	9/14
Perry et al., 2008 [26]	Fitness, motor competence and body composition as correlates of adolescent neck/shoulder pain: an exploratory cross-sectional study.	Neck/shoulder (posterior neck and upper trapezius)	Lifetime and one-month prevalence, pain duration more than 3 months	Cross-sectional	1608 (825/783) 14.1 ± 0.2	Upper and lower limb power (seated basketball throw, standing long jump) Trunk endurance (sustained back extension test, abdominal curls) Grip strength Shoulder flexibility (shoulder stretch) Motor competence (neurodevelopmental index)	Boys: Higher odds of neck pain when upper and lower limb power increased. Lower odds for neck pain when back muscle endurance reduced. R^2^ of models between 0.02 and 0.09. Girls: Higher odds for diagnosed neck pain when back endurance decreased or increased (U-shape) and abdominal endurance increased. Lower odds for neck pain when upper limb power increased and lower limb power and shoulder flexibility decreased. R^2^ of models between 0.001 and 0.06.	13/14
Straker et al., 2008 [28]	Sitting spinal posture in adolescents differs between genders, but is not clearly related to neck/shoulder pain: an observational study.	Neck/shoulder	Lifetime, one-month and point prevalence	Cross-sectional	1470 (713/757) 14.1 ± 0.2	Sitting spinal posture (photographs, 7 sagittal angles Questionnaire (lifetime, month, point prevalence of neck pain)	Adolescents with neck/shoulder pain: 2 degrees less trunk angle and 1 degree less cervicothoracic angle. After controlling for gender, no differences between the groups with and without neck pain.	9/14
Straker et al., 2009 [27]	Relationships between prolonged neck/shoulder pain and sitting spinal posture in male and female adolescents.	Neck/shoulder	Month prevalence, pain duration more than 3 months	Cross-sectional	1593 (814/779) 14.1	Sitting spinal posture (photographs, 7 sagittal angles Questionnaire (month prevalence of neck pain, duration of neck pain)	Adolescents with prolonged NSP: More flexed (decreased) cervicothoracic angle, more extended (decreased) trunk angle, more lordotic (decreased) lumbar angle, more anterior pelvic tilt. After controlling for gender: Association between prolonged NSP and increased lordosis/decreased lumbar angle (*R*^2^ = 0.02) and increased anterior pelvic tilt (*R*^2^ = 0.02).	11/14
Wirth et al., 2013 [29]	Spine Day 2012: spinal pain in Swiss school children - epidemiology and risk factors	Neck, thoracic spine, low back	Lifetime prevalence, recurrence, pain intensity, consequences (leisure activities, school absence, doctor visit, medication)	Cross-sectional	434 (211/223) 10.4 ± 2.8	Trunk functional stability (Matthiass test) Trunk asymmetry (forward bending test) Spinal mobility (fingertip-floor distance) Coordination (single leg stance)	No association of the outcome parameters with neck pain or mid back pain.	9/14

**Table 2:** Quality assessment of the included studies

Study	Study purpose	Literature	Design		Sample		Outcomes			Results				Data variability	Conclusions	Total score
			Study design	Bias (Y = 0, *N* = 1)	Description	Justification	Description	Reliability	Validity	Report of statistics	Appropriate analysis	Clinical importance	Dropouts	Estimates provided	Appropriate	
Cudre et al., 2006 [22]	Y	Y	Y	Y	Y	N	N/Y	N	N	Y/N	Y	N	NA	Y	Y	8
Dolphens et al., 2012 [23]	Y	Y	Y	N	Y	N	Y	Y	N	Y	Y	Y	NA	Y	Y	12
Dolphens et al., 2013 [24]	Y	Y	Y	N	N	N	Y	Y	N	Y	Y	Y	NA	Y	Y	11
Dolphens et al., 2014 [25]	Y	Y	Y	N	N	N	N	Y	N	Y	Y	Y	NA	Y	N	9
Perry et al., 2008 [26]	Y	Y	Y	N	Y	N	Y	Y	Y	Y	Y	Y	NA	Y	Y	13
Straker et al., 2008 [28]	Y	Y	Y	N	Y	N	Y	Y	N	N	Y	N	NA	Y	Y	9
Straker et al., 2009 [27]	Y	Y	Y	N	N	N	Y	Y	N	Y	Y	Y	NA	Y	Y	11
Wirth et al., 2013 [29]	Y	Y	Y	Y	N/Y	N	Y	N	N	Y	Y	Y/N	NA	Y	Y	9

The results of both reviewers are stated only where they differed

*Y* Yes, *N* No

### Physical risk factors

Mainly characteristics of the sitting [27, 28] and standing posture [23–25] were investigated as possible risk factors for NP, using a variety of angles. For clarification, the definitions of these angles are summarized in Table 3.

**Table 3:** Definitions of angles used to describe the sitting and standing posture

Angle	Lines forming the angle
Sitting posture [27, 28]	
Cervicothoracic angle	Line 1:SP T12 – SP C7
	Line 2:SP C7 – Tragus (ear)
Craniocervical angle	Line 1:SP C7 – Tragus (ear)
	Line 2:Tragus (ear) – Canthus (eye)
Head flexion	Line 1:Canthus (eye) - Tragus (ear)
	Line 2:Vertical line through Tragus (ear)
Lumbar angle	Line 1:Greater trochanter – ASIS
	Line 2:ASIS – SP T12
Neck flexion	Line 1:Tragus (ear) – SP C7
	Line 2:Vertical line through SP C7
Pelvic tilt	Line 1:Greater trochanter – ASIS
	Line 2:Vertical line through Greater trochanter
Trunk angle	Line 1:Greater trochanter – SP T12
	Line 2:SP T12 – SP C7
Standing posture [23–25]	
Body lean angle	Line 1:Lateral malleolus – SP C7
	Line 2:Vertical line through SP C7
Craniovertebral angle	Line 1:Tragus (ear) – SP C7
	Line 2:Horizontal line through SP C7
Pelvic displacement angle	Line 1:Lateral malleolus – Greater trochanter
	Line 2:Vertical line through Greater trochanter
Trunk lean angle	Line 1:Greater trochanter – SP C7
	Line 2:Vertical line through SP C7

*ASIS* anterior superior iliac spine, *SP* Spinous process

As for the sitting posture, two studies found an association between head position in relation to the thoracic spine (cervicothoracic angle) and NSP, when the model was not adjusted for gender [27, 28]. Head flexion, neck flexion and the craniocervical angle did not show any association with NSP (Table 4). Contrarily, increased lumbar lordosis (increased lumbar angle and pelvic tilt) was associated with prolonged NSP even after adjustment for gender [27], while adolescents with NSP sat with a slightly more extended trunk angle, but only when data were not adjusted for gender [27, 28]. In the standing posture, decreased anteroposition of the head (smaller craniovertebral angle) was associated with a decrease in odds for lifetime prevalence of NP in boys (OR = 0.95) and for seeking medical help for NP in girls (OR = 0.91), but the R^2^ values of the models were low (0.03 and 0.04, respectively) [23]. In contrast, sway-back posture was associated with NSP only in boys (lifetime prevalence: OR = 1.91, *R*^2^ = 0.02; 1-month prevalence: OR = 3.24, *R*^2^ = 0.04) [24], while the pelvic displacement angle did not show any association with NSP [23–25]. Furthermore, trunk asymmetry, functional stability and flexibility were not associated with neck pain [26, 29], while trunk endurance revealed some association that differed for boys and girls: boys with reduced back muscle endurance had lower odds for NSP (OR = 0.66), and girls had higher odds for NSP when back muscle endurance was either decreased (OR = 2.12) or increased (OR = 2.12) (U-shape) or abdominal muscle endurance was increased (OR = 1.57) [26]. The association of upper limb power and NSP was reversed for boys and girls: increased upper limb power was associated with higher odds for NSP in boys (OR = 2.47), but lower odds for NSP in girls (OR = 0.53). Lower shoulder flexibility was associated with lower odds for NSP only in girls (OR = 0.54) and grip strength did not show any association with NSP. Increased lower limb power was associated with higher odds for NSP in boys (OR = 3.47), while both, decreased (OR = 0.61) and increased (OR = 0.70) lower limb power, was associated with lower odds for NSP in girls. However, R^2^ values of these models were low (between 0.001 and 0.09, Table 1) [26]. General motor competence and single leg stance was not associated at all [26, 29].

**Table 4:** Results per outcome variable

Outcome variable		Changes in adolescents with NSP	No changes in adolescents with NSP
Sitting posture	Cervicothoracic angle	Slightly decreased in adolescents with NSP, if not adjusted for gender [28] Decreased in adolescents with prolonged NSP, if not adjusted for gender [27]	
	Craniocervical angle		Straker et al., 2008 [28]
			Straker et al., 2009 [27]
	Head flexion		Straker et al., 2008 [28]
			Straker et al., 2009 [27]
	Lumbar angle	More lordotic/smaller lumbar angle in adolescents with prolonged NSP after controlling for gender [27]	Straker et al., 2008 [28]
	Neck flexion		Straker et al., 2008 [28]
			Straker et al., 2009 [27]
	Pelvic tilt	Increased anterior pelvic tilt in adolescents with prolonged NSP after controlling for gender [27]	Straker et al., 2008 [28]
	Trunk angle	Decreased in adolescents with prolonged NSP, if not adjusted for gender [27] Slightly decreased in adolescents with NSP, if not adjusted for gender [28]	
Standing posture	Body lean angle	Sway back posture = large trunk lean angle and body lean angle in boys with neck pain [24]	Dolphens et al., 2012 [23]
			Dolphens et al., 2014 [25]
	Craniovertebral angle	Anteroposition of the head = smaller craniovertebral angle in boys with neck pain; more lifetime doctor visits in girls with anteroposition of the head [23]	
	Pelvic displacement angle		Dolphens et al., 2012 [23]
			Dolphens et al., 2013 [24]
			Dolphens et al., 2014 [25]
	Trunk lean angle	Increased trunk lean angle = increased posterior trunk tilt in boys with neck pain [23] Sway back posture = large trunk lean angle and body lean angle in boys with neck pain [24]	Dolphens et al., 2014 [25]
Trunk	Functional stability (Matthiass test)		Wirth et al., 2013 [29]
	Asymmetry (forward bending test)		Wirth et al., 2013 [29]
	Spinal mobility (fingertip-floor distance)		Wirth et al., 2013 [29]
	Endurance (sustained back extension test, abdominal curls)	Boys: lower odds for NSP when back muscle endurance reduced; girls: U-shape between NSP and back muscle endurance, higher odds for NSP when abdominal muscle endurance increased [26]	
Upper limb	Upper limb power (seated basketball throw)	Boys: higher odds for NSP when upper limb power increased; girls: lower odds for NSP when upper limb power increased [26]	
	Grip strength		Perry et al., 2008 [26]
	Shoulder flexibility (shoulder stretch)	Girls: lower odds for NSP when shoulder flexibility decreased [26]	
Other	Coordination (single leg stance)		Wirth et al., 2013 [29]
	Motor competence (neurodevelopmental index)		Perry et al., 2008 [26]
	Lower limb power (standing long jump)	Boys and girls: higher odds for NSP when lower limb power increased [26]	

*NSP* neck shoulder pain

Physical risk factors for MBP were sparsely investigated. The only parameter that showed some association with MBP in boys was increased posterior trunk tilt (increased trunk lean angle) in stance [23]. All other angles showed no association [23–25] as did the investigated trunk characteristics [22, 29].

## Discussion

An important finding of this review is that physical risk factors for adolescent NP and MBP are only sparsely investigated and the comparison of the studies is hindered by several factors. First, the definition of NP and MBP varied considerably. Some studies differentiated between NP and MBP [23–25, 29], while others focused on NSP [26–28] or upper back pain [22]. None of the studies provided a figure that illustrated the pain area(s) of interest. Second, sitting and standing posture were the main factors that were investigated, but the terminology used was confusing and inconsistent. The angle ‘neck flexion’ for example, used to describe head position in relation to the spinous process of C7 [27, 28], is the same as 90 degrees minus the ‘craniovertebral angle’ as used in other studies [23]. Using the same angles would considerably facilitate comparison between studies. These factors might explain why no distinct physical risk factors for adolescent NP and MBP emerged from this review.

### Physical risk factors for NP

A more lordotic sitting posture was associated with NSP after controlling for gender, but only if NSP was prolonged [27, 28]. An association between sitting posture and cervico-thoracic muscle activation was reported by Caneiro et al. [30], who observed an increased activity of the cervical erector spinae in slump sitting, albeit in adults. However, whether and how a more lordotic sitting position might affect neck muscle activity was not investigated. Nevertheless, correction of posture has been a recommended approach in the therapy of patients with dysfunctions of the cervical spine [31]. As for the standing posture, anteroposition of the head and sway-back posture were associated with NP measures, but predominantly in boys. However, the percentage of data variability explained by the corresponding statistical models was small (R^2^ between 0.03 and 0.06) [23–25]. Similarly, trunk muscle endurance showed some association with NSP, but the associations were different for boys and girls and of limited strength (R^2^ between 0.01 and 0.09) [26]. Thus, the association between posture and spinal pain might be sex-specific, although the reason for this is unclear [23]. In contrast, increased power of the lower limb was associated with higher odds of NP in both genders. A recent prospective study over two years reported that the 10% physically most active adolescents were at higher risk to develop spinal pain [32]. Thus presumably, lower limb power can be seen as a proxy measure for physical activity, which would explain this finding.

### Physical risk factors for MBP

Only five studies were found that investigated physical risk factors for MBP. This reflects the general observation that the thoracic spine receives remarkably less attention in the literature than the lower back or the neck [33]. That is why MBP was also named the stepchild of spinal research [34]. However, MBP is a common complaint in adolescence with a similar prevalence to LBP [3] that tends to persist into adulthood [10], where its one year prevalence is about 30% in the working population [3, 35]. Moreover, there are several studies in adults that underline the importance of the thoracic spine as basis for neck kinematics and for the development of neck pain [36–39], which is why addressing thoracic impairments in the management of cervical impairments was suggested [37]. Thus despite of some concerns for medicalization of MBP in adolescence [34], these findings underline the need for more research on epidemiology and risk factors for MBP and encourage particularly prospective studies in adolescents using a clear definition of MBP.

### Limitations

One limitation of this review is that it did not consider studies investigating NP and MBP in the context of physical activity, as this is mostly assessed via questionnaires. Nevertheless, a relation between respiratory parameters and thoracic spine mobility, neck muscle endurance and neck pain was observed [39, 40]. Furthermore, this review did not exclude studies that used a combination of neck and shoulder pain as done in a comparable review on physical load as risk factor for neck pain in adults [41]. This approach prevented from excluding some studies that actually fit the inclusion criteria of this review, but one should keep in mind that different factors might underlie pain in the proximal part of the upper arm and in the neck. Again, these two approaches reveal the need for a clear definition of neck pain.

## Conclusion

This systematic review could not identify distinct risk factors for adolescent NP and MBP. It could however show a strong need for prospective studies in this field using a consistent definition of NP and MBP, preferably using an illustration. The Young Spine Questionnaire (YSQ) [42] fulfills this requirement and its use is strongly encouraged, although further validation and cross-cultural adaptation is needed [1]. Furthermore, the inconsistency in reporting comparable outcomes should be reduced. This could possibly be achieved through an interdisciplinary consensus conference between stakeholders regarding this research topic and by further investigating the interplay between thoracic and cervical spine. Based on this review, sagittal alignment in sitting and standing should be further investigated as possible risk factors for adolescent NP and MBP using a consistent terminology for the outcomes and longitudinal research designs.

## Additional files

Additional file 1: Search strategy. (DOCX 508 kb)

Additional file 2: Quality assessment form and quality assessment of the included studies. (DOCX 35 kb)

Additional file 3: PRISMA checklist. (DOCX 37 kb)

## Abbreviations

LBP: Low back pain
MBP: Mid back pain
MeSH: Medical subheading
NP: Neck pain
OR: Odds ratio
TSP: Thoracic spine pain
YSQ: Young Spine Questionnaire
