# Supplementary material for: Physical risk factors for adolescent neck and mid back pain: a systematic review
Source: Chiropr Man Therap. 2018 Sep 24;26:36. doi: 10.1186/s12998-018-0206-y (PMC6151922; doi:10.1186/s12998-018-0206-y)
Supplement: Supplementary file 1 — Search strategy. (DOCX 508 kb) [file 12998_2018_206_MOESM1_ESM.docx]

**Additional file 1:** Search strategy

**Medline**

**
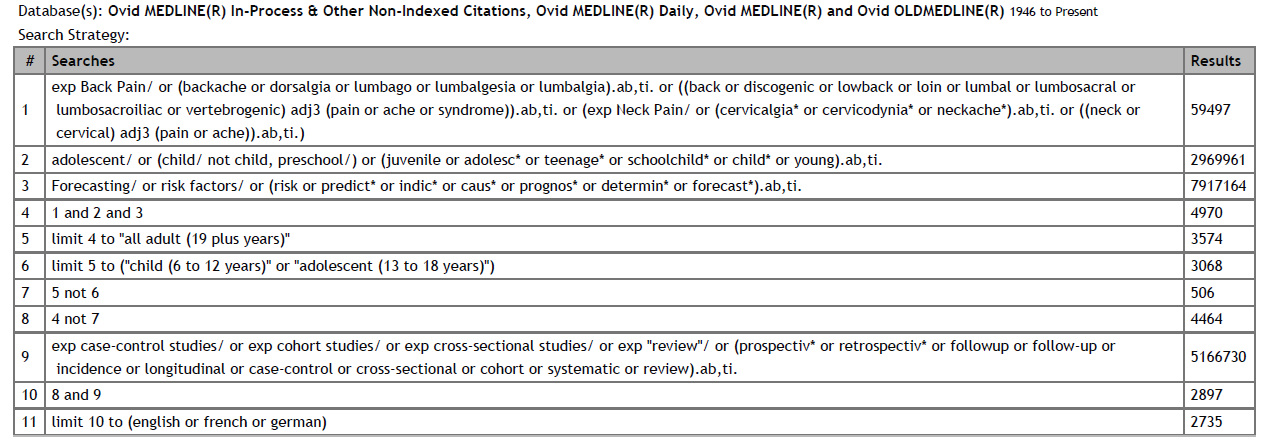
**

**Pubmed**

**
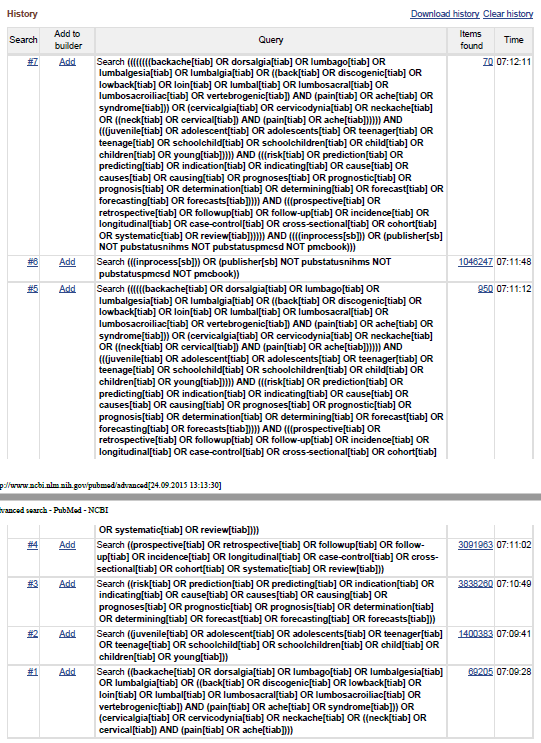
**

**Embase**

**
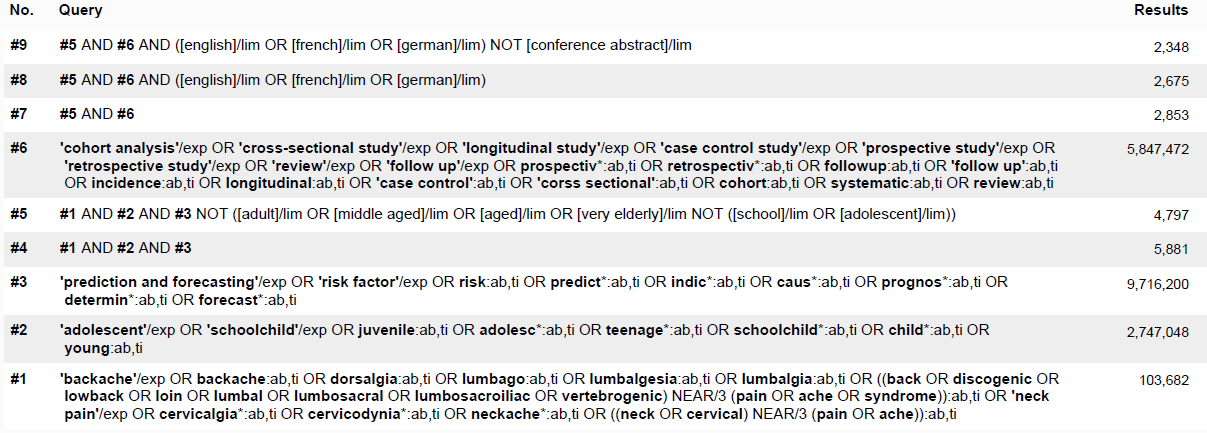
**

**Cinahl**

**
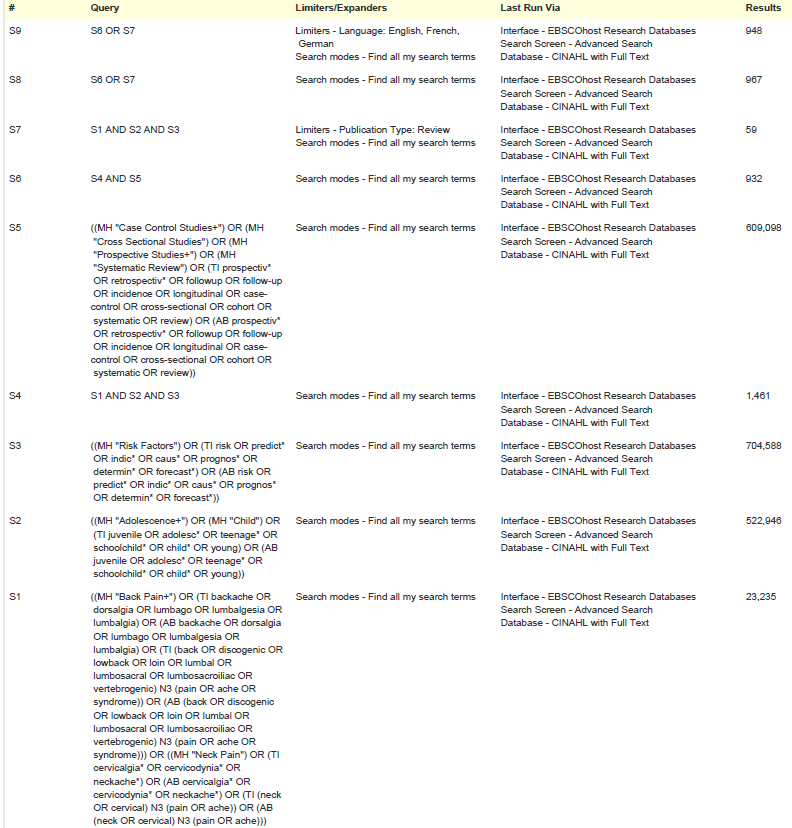
**

**Cochrane**

**
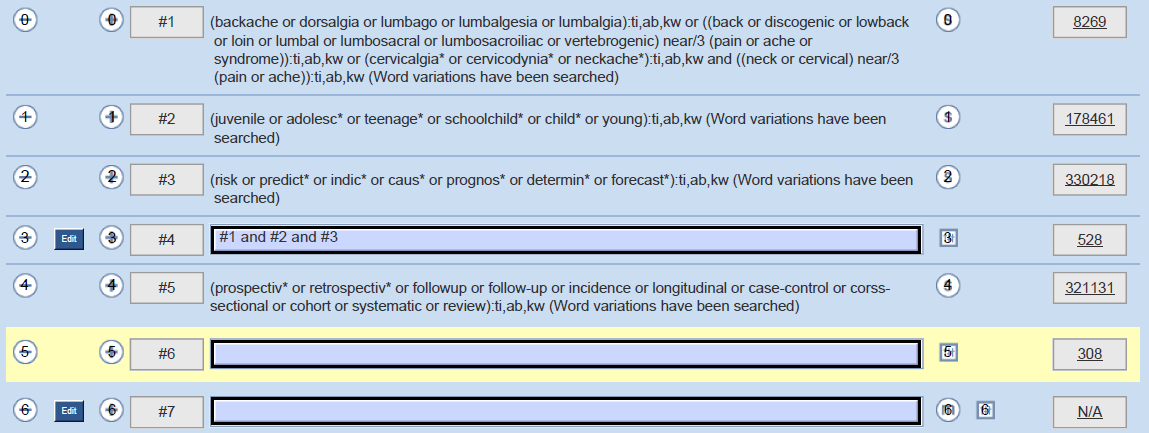
**

**PsychInfo**

**
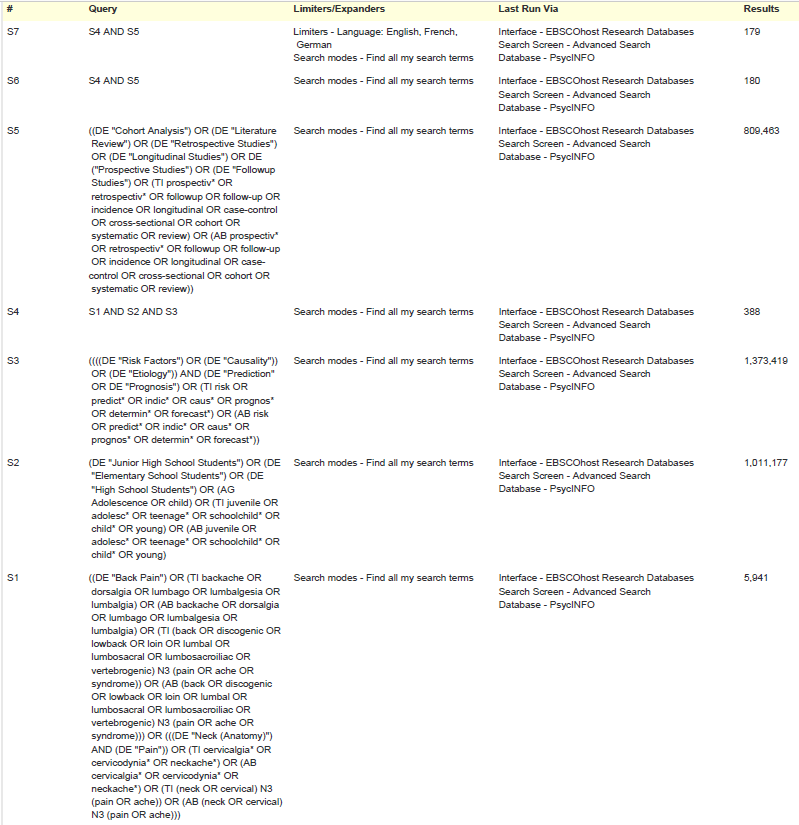
**

**Pedro**

| Back pain adolescen* | 14 records |
| --- | --- |
| Back pain child* | 47 records |
| Neck pain adolescen* | 19 records |
| Neck pain child* | 36 records |
| Pooled | 96 records |
