# Supplementary material for: Physical risk factors for adolescent neck and mid back pain: a systematic review
Source: Chiropr Man Therap. 2018 Sep 24;26:36. doi: 10.1186/s12998-018-0206-y (PMC6151922; doi:10.1186/s12998-018-0206-y)
Supplement: Supplementary file 2 — Quality assessment form and quality assessment of the included studies. (DOCX 35 kb) [file 12998_2018_206_MOESM2_ESM.docx]

**Additional file 2:** Quality assessment form and quality assessment of the included studies

Adapted from the “Critical appraisal form for quantitative studies” by Law et al [17] using the checklist by Downs and Black [19] and the form by Prins et al. [18]:

| **STUDY PURPOSE** | Was the purpose stated clearly? | Yes/No |
| --- | --- | --- |
| **LITERATURE** | Was relevant background literature reviewed? | Yes/No |
| **DESIGN** | Was the design appropriate for the study question? | Yes/No |
|  | Were there any biases (random/nonsystematic error or measurement bias/systematic error) that may have influenced the results (apart from reliability and validity of the outcomes)? | Yes/No |
| **SAMPLE** | Was the sample described in detail? | Yes/No |
|  | Was sample size justified? | Yes/No |
| **OUTCOMES** | Were the methods of outcome measurement described sufficiently? | Yes/No |
|  | Were the outcome measures reliable? | Yes/No |
|  | Were the outcome measures valid? | Yes/No |
| **RESULTS** | Results were reported in terms of statistical significance? | Yes/No |
|  | Were the analysis method(s) appropriate? | Yes/No |
|  | Clinical importance was reported? | Yes/No |
|  | Drop-outs were reported? | Yes/No/Not applicable |
| **DATA VARIABILITY** | Does the study provide estimates of the random variability in the data for the main outcomes? | Yes/No |
| **CONCLUSIONS AND IMPLICATIONS** | Conclusions were appropriate given study methods and results? | Yes/No |

TOTAL: Maximal 14 points for cross-sectional studies and 15 points for prospective studies
